# Supplementary figures and images for: The use of virtual reality in studying prejudice and its reduction: A systematic review
Source: PLoS One. 2022 Jul 14;17(7):e0270748. doi: 10.1371/journal.pone.0270748 (PMC9282653; doi:10.1371/journal.pone.0270748)

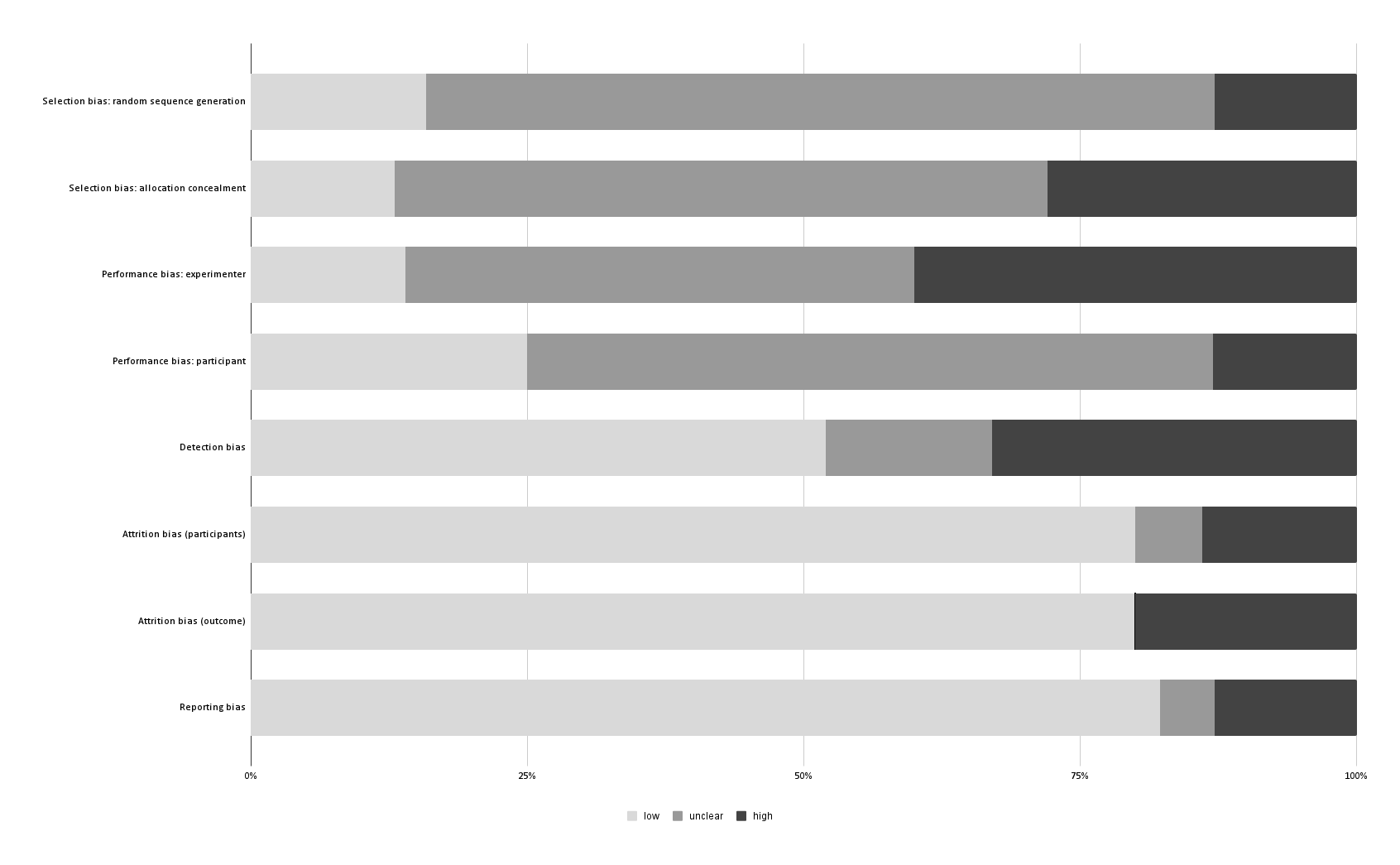

Supplement: S1 Appendix — (TIF) [file pone.0270748.s002.tif]
