# Supplementary material for: The use of virtual reality in studying prejudice and its reduction: A systematic review
Source: PLoS One. 2022 Jul 14;17(7):e0270748. doi: 10.1371/journal.pone.0270748 (PMC9282653; doi:10.1371/journal.pone.0270748)
Supplement: S2 Appendix — (PDF) [file pone.0270748.s003.pdf]

The use of virtual reality in studying prejudice and its reduction: a systematic review – Database search strategy

Controlled vocabulary terms have been used for database searches. We selected the search terms through the *PsycINFO Thesaurus of Psychological Index Terms*.
The terms selection process initially resulted in 14 controlled vocabulary terms, of which 7 are related to virtual reality, and the remaining 7 to intergroup attitudes.

The searches were re-run with updated terms on 10.01.2022 following reviewers' comments.
We removed duplicates between the old and new search using the method proposed by Bramer & Bain (2017) https://www.ncbi.nlm.nih.gov/pmc/articles/PMC5490709/ which resulted in 1873 additional hits from the 2021 search to the 2022 search.

Below are listed the database-specific strings used and the dates of entry.

Web of Science

search string 1 (searched 07/01/2021):
((TS = ((Vr OR virtual reality OR immersive virtual environment OR simulation-based assessment OR virtual reality exposure therapy OR virtual OR augmented reality) AND (intergroup relations OR ingroup outgroup OR prejudice OR discriminat* OR bias OR stereotyp* OR stigma*))))

6356 citations retrieved.

search string 2 searched (10/01/2022):

((TS = ((Vr OR virtual reality OR immersive virtual environment OR simulation-based assessment OR virtual reality exposure therapy OR virtual OR augmented reality)

AND

(intergroup relations OR ingroup outgroup OR prejudice OR discriminat* OR bias OR stereotyp* OR stigma* OR intergroup attitude* OR outgroup attitude*)

)))

7380 citations retrieved.

Scopus

search string 1 (searched 07/01/2021):
TITLE-ABS-KEY ((*vr* OR *"virtual reality"* OR *"immersive virtual environment"* OR *"simulation-based assessment"* OR *"virtual reality exposure therapy"* OR *virtual* OR *"augmented reality"*) AND (*"intergroup relations"* OR *"ingroup outgroup"* OR *prejudice* OR *discriminat** OR *bias* OR *stereotyp** OR *stigma**)) AND (LIMIT-TO (LANGUAGE , *"English"*) OR LIMIT-TO (LANGUAGE , *"German"*) OR LIMIT-TO (LANGUAGE , *"Italian"*))

7070 citations retrieved.

search string 2 searched (10/01/2022):
TITLE-ABS-KEY ((vr OR "virtual reality" OR "immersive virtual environment" OR "simulation-based assessment" OR "virtual reality exposure therapy" OR virtual OR "augmented reality") AND ("intergroup relations" OR "ingroup outgroup" OR prejudice OR discriminat* OR bias OR stereotyp* OR stigma* OR "intergroup attitude*" OR "outgroup attitude*")) AND (LIMIT-TO (LANGUAGE , "English") OR LIMIT-TO (LANGUAGE , "German") OR LIMIT-TO (LANGUAGE , "Italian"))

8027 citations retrieved.

Psycinfo

search string 1 (searched 07/01/2021):
((Vr or "virtual reality" or "immersive virtual environment" or "simulation-based assessment" or "virtual reality exposure therapy" or virtual or "augmented reality") and ("intergroup relations" or "ingroup outgroup" or prejudice or discriminat* or bias or stereotyp* or stigm*)).mp. [mp=title, abstract, heading word, table of contents, key concepts, original title, tests & measures, mesh]

1376 citations retrieved.

search string 2 searched (10/01/2022):
((Vr or "virtual reality" or "immersive virtual environment" or "simulation-based assessment" or "virtual reality exposure therapy" or virtual or "augmented reality") and ("intergroup relations" or "ingroup outgroup" or prejudice or discriminat* or bias or stereotyp* or stigm* or "intergroup attitude*" or "outgroup attitude*")).mp. [mp=title, abstract, heading word, table of contents, key concepts, original title, tests & measures, mesh word]

1559 citations retrieved.
