# Supplementary material for: The use of virtual reality in studying prejudice and its reduction: A systematic review
Source: PLoS One. 2022 Jul 14;17(7):e0270748. doi: 10.1371/journal.pone.0270748 (PMC9282653; doi:10.1371/journal.pone.0270748)
Supplement: Attachment — Submitted filename: response to reviewers.docx [file pone.0270748.s004.docx]

**Response to reviewer comments**

**Reviewer #1**: Title: The use of virtual reality in studying prejudice and its reduction: a systematic review

The present review illustrates VR studies published to date on social attitudes and stereotyping with the goal of highlighting the contributions that VR techniques can offer in this field as well as the limitations of the current work.

The goal of this review is very interesting and timely since the use of VR techniques is rapidly growing in social psychology research. The VR techniques indeed represent a promising (but still under-investigated instrument) in social research concerning intergroup dynamics.

However, I have some reservations about the organization, rationale, and theoretical background of this review that prevent me from supporting its publication in the present form (see my comments/suggestions to authors).

Introduction:

Authors do not provide a background of the topic. This review is on social attitudes and stereotypes, and their malleability. I'm actually quite surprised to see that the reference list includes only a few studies from a Social Psychology or Social Cognition journal, despite the fact that this field has been studying the topic for several decades. I'm not trying to be a gatekeeper here as I recognize that many fields beyond Social Psychology/Cognition are interested in this topic. But you can't simply ignore 30+ years of scholarship. What do we know about social attitudes and stereotyping? How can we assess them? How do they operate? How can they be changed?

We thank the reviewer for pointing out the overlook of the theoretical and methodological advances in a study on intergroup attitudes and prejudice in social psychology, which compromised the development of  a solid foundational background of this study. However, as being social psychologists ourselves, we indeed agree that this is critical not only in terms of presenting the point of departure for the readership but also for the success of the review to describe and critically evaluate the research on intergroup contact and attitudes in VR. We have now provided a better theoretical overview of the concepts studied with more references to social psychological literature on the topic.

Similarly, I suggest introducing and describing some key concepts and terms that are essential to understand the topic (e.g., social groups, attitude, stereotype, intergroup relations, group membership, in-group and out-group members). As regards the terms included in the present version of the manuscript, I have some reservations about the use of the term “prejudice”. The term “prejudice” typically refers to negative evaluations that may be preconceived and consciously experienced. The studies reported in this review included beliefs (stereotypes) and evaluations with some degree of favor or disfavor (attitudes) about social groups assessed by using different paradigms (e.g., explicit and implicit measures). I would suggest the authors to substitute the term “prejudice” with the terms attitudes and stereotypes.

Again, we agree that some key concepts were not introduced sufficiently and have now elaborated on this. We also agree that the need for more specificity particularly relates to the concept of prejudice in relation to other concepts such as intergroup/outgroup (implicit/explicit) attitudes, ingroup/intergroup bias and intergroup/group stereotypes and emotions. Though often being studied and operationalised interchangeably, these concepts nevertheless refer to different components and forms of prejudice and they have been studied with different measures, which on their own part, also sets the limits for studying and postulating prejudice, as noted by the reviewer in the case of IAT measures used to study implicit bias. In the revised version of the manuscript, we not only put efforts in explaining the differences between the concepts and measures but also pay closer attention to the used concepts and measures in evaluating the results of the reviewed studies.

In addition, I suggest the authors to better highlight the motivation for using VR in this field. Over the past decades, several studies have attempted to develop interventions (e.g., see Dasgupta and Greenwald, 2001; Dasgupta, DeSteno, Williams, & Hunsinger, 2009; Legault, Gutsell, & Inzlicht, 2011; Mann & Kawakami, 2012; Lai et al. 2014, 2016) or used specific techniques (e.g., Marini et al. 2018) to produce changes in attitudes and stereotypes. How VR can help us to understand social interactions? Surely there is a valid motivation for doing this, right? What important gap in the social attitudes and stereotypes literature does this project fill? By providing the relevant literature on this topic, you might specify this gap to the readers in order to understand the potential and (maybe) unique contribution that VR can provide.

We agree with the reviewer’s comment that the motivation to use VR in the field of intergroup processes is not self-evident despite the rapid development of VR technologies and the increase in intervention studies using this environment. We now have added sections to the introduction that make clear why VR has been seen as well-suited for this field of research and how it relates to and expands other lines of prejudice research.

Results:

Authors illustrate the contribution of VR by considering the type of contact, stigma, presence of mediators, and moderators in the different studies. I think that authors should also create a section in which they compare the results found using implicit and explicit measures of attitudes/stereotypes or other instruments to assess relevant psychological constructs (e.g., empathy) in social relationships. It is important for the readers to be able here to identify the results obtained using different outcome measures. At the moment, these results are not clearly presented in the manuscript.

Upon revising our manuscript, following the recommendation of Reviewer 2, we have now shifted the focus of the results section to what we consider key high quality studies in this field and report their results in more detail (as opposed to other studies that suffer from serious methodological problems). This includes an enhanced focus on effects on different kinds of outcomes, as proposed by the reviewer.

Conclusion and Discussion:

Please clarify also the effects of VR based on the outcome measures used in the study. What are the conclusions about implicit measures? What are the conclusions about explicit measures? What about the other paradigms used in these studies?

The discussion section now provides more clarification on the different studied outcomes, also distinguishing the effects on implicit from those on explicit measures, and again, with a special focus on key studies in the field.

Minor points:

- Page numbers are missing.

Page numbers have been added to the manuscript.

- Please provide a description of the experimental paradigms mentioned in the text (e.g., shooting paradigm, Implicit Association Test).

In the original version of the manuscript, we described the IAT in paragraph 4.1. In the revised version, we have now introduced the IAT in the theoretical background section, and provide a short description of the shooter bias in result section 4.2.2.

- Please provide also in the main manuscript the main terms of research used in this review. I think that would have been appropriate include in your search also the term “attitude” and the most used instruments in this field (e.g., Implicit Association Test).

The text now includes a footnote with the search terms. We agree that the term “attitude” is important. However, including the term “attitude” would have led to an insurmountable amount of search results unrelated to the field of intergroup relations. We therefore expanded our search with the terms “outgroup attitude” and “intergroup attitude”. This brought 19 new studies in our review.

- Page 19 (?): Please consider some additional studies about the relation between implicit-explicit measures (e.g., Nosek and Smyth, 2007; Nosek et al., 2007; Greenwald and Nosek, 2008; Cunningham et al. 2004)

Obviously, the relation between implicit and explicit measures is a very debated issue, and we do agree that a more in-depth discussion of the differences between the implicit measures based on association strengths and explicit measures reflecting normative evaluative processing is in place in order to better understand the potentially different effects of VR contact in different studies and the potential on VR in studying and shaping implicit and explicit attitudes. We thank for the suggested references as they provide important arguments to this discussion (see paragraphs 1.1, 4.2.1, and 5.1).

- Page 21(?): “Nevertheless, some contrasting evidence shows that embodying black avatars can

also lead to worsened implicit attitudes, depending on the social context in which the experience takes place (Banakou et al., 2020)”

Can you clarify this claim in the text? Which social contexts were examined in this study? Which social context worsened implicit attitudes?

This study is now discussed in more detail in paragraphs 4.2.1 and 4.2.2, as we regard it to be one of the key studies in this field.

**REVIEWER #2**:

Let me begin by saying that I learned something from reading this manuscript and I don’t have a lot to criticize. My summary is simple (though I’ll elaborate): I’m not certain that this is an area of investigation that is ready yet for a meta-analysis. In brief, the manuscript reads more like a very thorough Introduction than a meta-analysis. Often times, small groupings of results are pitted against one another such as in the following section (p. 19): “Similar encouraging results have been found when measuring explicit attitudes, as shown by Christofi et al. (2020) and Tong et al. (2020, 2017). However, other studies contest these findings by showing that intergroup contact experienced from the minority perspective doesn’t necessarily have any effect on intergroup attitudes (see Hasler et al., 2016; Oh et al., 2016; Starr et al., 2019).” This, to me, reads like an Introduction wherein the author(s) would include their preferred interpretation as well as a “but see” section of the citation. That said, Section 4.3 (Methodological issue and advantages) begins to outline what remains to be done. In so doing, this manuscript could serve well as a summary of the state of the field as well as an initial attempt at setting up the next couple of years of research. The quibble that the outline may be premature by those couple of years is obviously based on a somewhat arbitrary decision (i.e., exactly how many studies are needed before we are ready for a meta-analysis?). In the end, I found this manuscript more useful in terms of how it highlights what hasn’t been done. That is, of course, somewhat atypical for, as the title implies, a “systematic review”. As a reader, I might feel as though I had not gotten what I came for. I wonder if a change in title might be in order. Something like “a systematic review of the early research returns and an outline for the near future”. Something that both conveys that 1) there is not a lot of research completed yet (and so the conclusions will be relatively few and tenuous) and 2) the reader will get an organized view of what needs to happen next. To that end, I think the authors could spend a bit of time focusing current section 4.3 toward future directions and adding a bit more about what is needed. In that way, in my opinion, this would become something that anyone interested in entering into this subarea should read.

We thank the reviewer for their thoughtful comments on the manuscript and would first like to clarify that this article does not attempt to provide a meta-analysis in the sense of statistically weighing the evidence - we agree that research in this field is too under-developed and unsystematic for this to be done in a meaningful way. Instead, we aimed at providing a systematic review of the research on VR contact and intergroup attitudes conducted so far in order to describe the different lines of research, the designs and measures used, and the effects obtained. We also agree that the way we described our results in the original version of the manuscript was somewhat dissatisfying as  they did not draw a clear picture on whether VR interventions are effective or not. We agree with the reviewer that this was due to the equal weight given to the reviewed studies neglecting the clear differences in their quality. In our revision, we now attempt to alleviate this by giving more emphasis to studies that we consider to be of high methodological quality. This way, we, to some degree, avoid the ambiguity created by seemingly assigning equal weight to methodologically heterogeneous studies and manage to come to somewhat clearer conclusions.

The reviewer writes that “this manuscript could serve well as a summary of the state of the field as well as an initial attempt at setting up the next couple of years of research” - this is exactly what we are trying to do and we hope that our revisions lay this out more clearly.

Finally, what is clear is that the field of research undergoes very rapid development, which is evident in the revised manuscript. Namely, the update of the search conducted for the original version of the manuscript has brought 19 new studies in our review published after our initial submission in 2021. The increased data-base also allowed us to draw better overview of and conclusions from the results obtained in the field.
