# Supplementary material for: The use of virtual reality in studying prejudice and its reduction: A systematic review
Source: PLoS One. 2022 Jul 14;17(7):e0270748. doi: 10.1371/journal.pone.0270748 (PMC9282653; doi:10.1371/journal.pone.0270748)
Supplement: Attachment — Submitted filename: response_to_reviewers.docx [file pone.0270748.s005.docx]

**Response to reviewer comments**

**Reviewer #1**: Title: The use of virtual reality in studying prejudice and its reduction: a systematic review

I think the present version of the manuscript has improved compared to the previous one. However, I still have some concerns that prevent me from supporting its publication. Unfortunately, the authors did not fully address some crucial points raised in my previous review.

We are sorry to read that reviewer 1 did not find the previous version of the manuscript as satisfying, but it is good to know that they appreciated the volume and direction of changes made. In the current revision, we did our best to respond and correct every remaining detail of their critical comments, which, in our view, again clearly improved the potential contribution of the study.

Here are some examples.

I suggested the authors to provide a background of the topic that can summarize what we know about the malleability of attitudes and stereotypes. This piece of information is still lacking in the text. In the revised manuscript, the authors only describe in more detail one kind of intervention developed to change social attitudes and stereotypes (i.e., intergroup contact). Again, you cannot ignore 30+ years of scholarship on this topic. Over the past decades, several studies have developed interventions to change attitudes and stereotypes (e.g., see Dasgupta and Greenwald, 2001; Dasgupta, DeSteno, Williams, & Hunsinger, 2009; Legault, Gutsell, & Inzlicht, 2011; Mann & Kawakami, 2012; Lai et al. 2014, 2016; Marini et al. 2018). One or two paragraphs on this topic are necessary for the readers to understand what we know in this field and how VR may be useful. By reading the introduction of this manuscript, it seems social psychologists used only interventions based on intergroup contact to modulate social attitudes and stereotypes. I know that intergroup contact was the most used intervention in the VR studies, but this does not imply that this intervention is the only one that can be implemented using VR or the only one that deserves to be described. On this latter point, please also consider some recent studies on implicit bias showing that intergroup contact and perspective-taking interventions are not the most effective interventions to modulate social attitudes and stereotypes (see, for example, Lai et al. 2014; Lai et al. 2016).

The readers deserve to know that, over the years, many experimental interventions have been developed in this research field. The background provided by the authors is too limited.

We thank the reviewer for spelling out this comment and fully agree that there was space and function for the suggested addition. In light of the reviewer’s comment, we added three paragraphs on the malleability of intergroup attitudes to provide an accurate overview about the development of intervention research in the area (see section 1.2. Malleability of intergroup attitudes). To be able to cover the volume of intervention research, we decided to focus on meta-analytic studies. Thus, in the first paragraph, we describe Paluck and Green’s (2009) classification of social scientific and psychological interventions designed to influence intergroup bias. In paragraphs 2 and 3, we discuss the malleability of implicit racial prejudice based on the results of the meta-analyses of Lai et al. (2014, 2016), followed by the results of Beelmann and Heinemann’s (2014) meta analysis of different structured programs to reduce explicit prejudice and negative intergroup attitudes. Only then do we move on to discussing the interventions based on contact theory.

In addition, a crucial aspect that was not addressed is the effectiveness of VR on implicit and explicit measures. What do we know about that? Is VR more effective on the implicit or explicit bias? Are the results of the reviewed studies unclear on this topic? This issue is something that the authors need to raise in their review, considering that attitudes and stereotypes assessed by implicit measures have shown to predict behavior more accurately than explicit measures in some specific domains. This aspect is relevant because the final goal of reducing attitudes and stereotypes is to reduce their effect on behavior.

Upon revising our manuscript, we have now added a paragraph in the “Results” section to outline differences in findings regarding the effect of VR contact on intergroup bias assessed using implicit and explicit measures. Based on our analysis, we now conclude that although results are somewhat inconsistent for both measures and depend on the type of intergroup contact studied (majority vs minority perspective), “It seems like implicit measures unveil potential effects of VR-based interventions that might not appear in explicit measures of intergroup attitudes” (p. 27). By making this conclusion, we agree with the reviewer that this is an important conclusion to be made which does not only highlight the potential of VR contact to improve (implicit) attitudes but also outlines its potential to change intergroup behaviors. Furthermore, we have expanded those findings further in the discussion.

Again, I still have some concerns about using the term “prejudice” in this manuscript as it includes studies in which they used implicit measures as the IAT. I believe this term is not appropriate given the long debate in the literature about what implicit measures assess.

We understand this concern. We have taken care of this point by describing the way the concepts of prejudice, intergroup attitudes and intergroup bias are separate but interrelated and how they have been used in different literatures to pinpoint the specific nature of the phenomena studied. We also carefully went through the text and checked that we refer to prejudice only when we talk about the general area of prejudice reduction intervention research. In contrast, we replaced “prejudice” with “intergroup bias” each time we discuss implicit measures. We hope that this way we can do both: speak to the readership about the importance of prejudice reduction and to acknowledge different theoretical and methodological approaches to study intergroup phenomena.

Finally, please report the correct references when you cite an instrument. The appropriate citation for the IAT is not Greenwald & Banaji (1995) but Greenwald, McGhee, and Schwartz (1998).

We corrected this unintended error in referencing.

**Reviewer #2**: Title: The use of virtual reality in studying prejudice and its reduction: a systematic review

I read the revised manuscript with interest and pleasure. I feel as though the review process made this paper quite a bit stronger. Particularly as a result of the authors’ openness to doing a lot of additional work in response to reviewer requests. And, as the authors note in their response to the reviews, the current version is also strengthened by the number of additional papers that could now be included – the number of which serves to indicate how important the topic is. This is a paper that I will send to my students as a way to understand the state of a topic. That’s a rather self-centered compliment, but it’s the best way I know to indicate a paper’s potential utility.

I note that it will be worthwhile to do one more very slow read through for typos and things related to some annoying idiosyncrasies of the English language, particularly related to verb tenses and singular vs. plural formations.

We are pleased to read that reviewer #2 considers our work ready for publication, and we wish to thank for the good words on the meaningfulness of the manuscript. We have now proofread the manuscript to check for typos and enhance linguistic consistency. We hope that this and the additional revisions have further strengthened the manuscript.
